# Effect of cognitive-behavior therapy for children with functional abdominal pain: a meta-analysis

**DOI:** 10.1186/s12876-024-03120-2

**Published:** 2024-02-03

**Authors:** Xiaolan Huang, Nan Jia, Yan Zhang, Yanyan Hao, Fei Xiao, Chunrong Sun, Xiaodai Cui, Fei Wang

**Affiliations:** 1 Affiliated Children’s Hospital of Capital Institute of Pediatrics, Beijing, 100000 China; 2 Experiment center, Capital Institute of Pediatrics, Beijing, China

**Keywords:** Cognitive-behavior therapy, Functional abdominal pain, Children, Meta-analysis

## Abstract

**Background:**

Cognitive-Behavior Therapy (CBT) is the validated non-pharmacological treatment for chronic pain in pediatric patients. While some suggested CBT were comparable to the usual care in reducing children’s functional abdominal pain. This meta-analysis was designed to systematically review the literature for RCTs that investigated the efficacy of CBT in children with functional abdominal pain (FAP).

**Methods:**

PubMed, Embase, and the Cochrane library were searched for papers published up to October 2022. Studies applying different CBT delivery methods (in-person, web-based, phone-based) were included in this meta-analysis to evaluate the comprehensive effectiveness of CBT compared with usual care. Weighted and standardized mean difference with the 95% confidence intervals were used for the synthesis of the results. Primary outcome was the decrease of functional disability inventory (FDI) and the secondary outcomes were the decrease of severity in pain intensity, depression, anxiety, gastrointestinal symptoms, and improvement in physical quality of life (QoL).

**Results:**

A total of 10 RCTs with 1187 children were included in the final analysis. The results showed that CBT resulted in better effect in reducing functional disability inventory (SMD=-2.282, 95%CI: -4.537 to -0.027, *P* = 0.047), pain intensity (SMD=-0.594, 95%CI: -1.147 to -0.040, *P* = 0.036), and improving QoL (SMD = 14.097, 95%CI: 0.901 to 27.292, *P* = 0.036) compared with the control groups. Comparable effects were observed in the severity of depression (SMD=-0.493, 95%CI: -1.594 to 0.608, *P* = 0.380), anxiety (SMD=-0.062, 95%CI: -0.640 to 0.517, *P* = 0.835), and gastrointestinal symptoms (SMD=-1.096 95%CI: -2.243 to 0.050, *P* = 0.061) between CBT and usual treatment.

**Conclusions:**

We observed the differences in post-treatment FAP and pain intensity for children receiving CBT compared with children receiving treatment as usual. CBT in the setting of FAP demonstrates promising developments and highlights the need for future research.

**Supplementary Information:**

The online version contains supplementary material available at 10.1186/s12876-024-03120-2.

## Background

Functional abdominal pain (FAP) is a highly prevalent gastro-intestinal disorder which can lead to significant impairments of functioning among children, affecting 14% of youth worldwide [1, 2]. The abdominal symptoms of pediatric FAP can be persistent and remain into adulthood, and are not attributed to other medical conditions [3, 4]. Specifically, youth with FAP often experience long-term pain-related distress [5] and functional impairment, even after extensive healthcare utilization [6]. Despite that recent advancements in the understanding of FAP in children are reflected in the evolution of diagnostic criteria, limited treatments such as dietary and pharmacological interventions [7, 8] have demonstrated comparable effect over placebo. There are some evidence [9–12] suggesting cognitive behavioral therapy (CBT), which typically focusing on cognitive restructuring of maladaptive thoughts, exposure exercise, and relaxation, can decrease pain, disability, and increase quality of life. More studies are currently mounting to clarify which treatment components of CBT are effective, and, through which mechanisms to effectively benefit which type of patients.

To date, CBT [13] is deemed as the a well-validated non-pharmacological treatment for chronic pain in clinical use, demonstrating significant effect in the treatment of chronic and recurrent pain, such as headaches, gastro-intestinal, musculoskeletal and disease related pain. One of the primary goals of CBT is to identify and correct cognitive distortions and maladaptive behavior, which may involve patient and parental beliefs about the child’s illness and factors such as activity restriction, school attendance and social involvement [14]. Despite a given number of recent randomized controlled trials (RCTs) have published to investigate the connection between children’s functional abdominal pain and CBT, still, inconsistent results were noted across studies. Some claimed CBT can be significantly effective in reducing children’s functional abdominal pain, increasing child coping skill, showing improvements in anxiety symptoms, as well as improving the quality of life compared to the usual care [10, 15, 16]. While some [17, 18] suggested CBT were comparable to the usual care. Given the increasingly recognition of psychological factors, including anxiety and depression, as well as stress conditions and quality of life in the contribution of FAP in children [19], this meta-analysis was aims to systematically review the literature for RCTs that explored the efficacy of CBT in addressing not only the physical symptoms of FAP but also in mitigating the associated psychological distress such as depression, anxiety, and quality of life, which intimately linked with the manifestation and severity of FAP and influenced the overall well-being and daily functioning of affected children.

## Methods

### Ethical statement

We developed the framework of the current systematic review and meta-analysis according to the recommendations issued by Cochrane Collaboration for the purpose of ensuring the methodological quality because we did not register formal protocol [20]. We did not impose ethical approval and patients’ informed consent because all essential data in the current systematic review and meta-analysis was extracted from published studies.

### Literature search

This meta-analysis was conducted according to the Preferred Reporting Items for Systematic Reviews and Meta-Analyses (PRISMA) guidelines [21]. The relevant articles were searched using the PICOS principle [22], followed by screening on the basis of inclusion and exclusion criteria. PubMed, Embase, and the Cochrane library were searched for available papers published up to October 2022, using the MeSH terms ‘Cognitive Behavioral Therapy’, ‘Child’, ‘Pediatrics’, Adolescent’, and ‘Abdominal Pain’, as well as relevant key words.

The eligibility criteria were: (1) population: children who were diagnosed with FAP according to established gastroenterologist-determined criteria; (2) interventions: CBT with or without clinical standard treatments; (3) control: standard treatments (including medical treatment or education); (4) study type: RCTs; and (5) language limited to English.

### Data extraction

Two experienced investigators (Xiaolan Huang and Fei Wang) independently assessed the studies using the inclusion/exclusion criteria. The data were extracted by the two investigators independently, using a pre-specified protocol. The characteristics of each study, including authors, year of publication, country of origin, study design, disease type, and sample size, were compiled. The treatment parameters included the type of intervention, detailed description of the CBT, and the duration of CBT. Other parameters included the assessment tools for the outcomes, and the follow-up visits of the assessment. The primary outcome were the measurements of abdominal functional pain, defined by the functional disability inventory (FDI) [23]. FDI is a 15-item measure of difficulty in performing activities, which has been proven valid for youth with functional abdominal pain [24]. Pain intensity and severity was defined by a combination of the Visual Analog Scale (VAS), Faces Pain Scale-Revised (FPSR), Abdominal Pain Index (API), and the Numerical Rating Scale (NRS). The assessments of depression and anxiety was defined by a combination of the Children’s Depression Inventory (CDI), Bath Adolescent Pain Questionnaire (BAPQ), and Revised Child Anxiety and Depression Scale (RCADS). The severity of gastrointestinal symptom was assessed by the Children Somatization Inventory (CSI). The quality of life was defined by Pediatric Quality of Life Inventory (PedsQL).

### Quality of the evidence

The level of evidence of all included studies were assessed independently by two authors using the RoB-2 criteria [25]. The included randomized controlled studies were assessed respectively in five domains regarding the (1) Bias arising from the randomization process; (2) bias due to deviations from intended interventions; (3) bias due to missing outcome data; (4) bias in measurement of the outcome; and (5) bias in selection of the reported result. Finally, an overall evaluation was concluded per study based on the assessments of each individual aspect. Discrepancies in the assessment were resolved through discussion until a consensus was reached. (Additional file 1)

### Data synthesis

The measurements for the pain intensity/severity and the depression/anxiety varied among the studies. The standardized mean difference (SMD) was used in the analysis to avoid bias per the Cochrane Handbook suggested. If a trial compared different types of CBT with the control group, then the children in the control group were equally divided between treatment groups to prevent control participants from being counted more than once. Each set of data was entered as a separate trial. The parameters were extracted during the last follow-up period reported. For studies that did not present their results as means ± standard deviations, the results were estimated based on the reported parameters (median, standard error, IQR or 95%CI) [26], which was also advised in the Cochrane Handbook [20].

### Statistical analysis

STATA SE 14.0 (StataCorp, College Station, Texas, USA) was used for all analyses. The effects and corresponding 95% confidence intervals (CIs) were used to compare the outcomes. The heterogeneity across the included studies was calculated using the I^2^ and Q statistic. Random-effect model was applied to investigate the effect of CBT on the post-treatment measurements, as we realized the RCTs included in our meta-analysis applied different strategies performing the cognitive-behavior training and used different tools in assessing the outcomes, which can lead to great heterogeneity in terms of the intended intervention and measurements of outcomes. We didn’t assess the potential publication bias by funnel plots and Egger’s test, because the number of studies included in every meta-analysis were small, in which case the funnel plots and Egger’s test could yield misleading results and were not recommended [20, 27]. 

## Results

### Selection of the studies

**Fig. 1:**
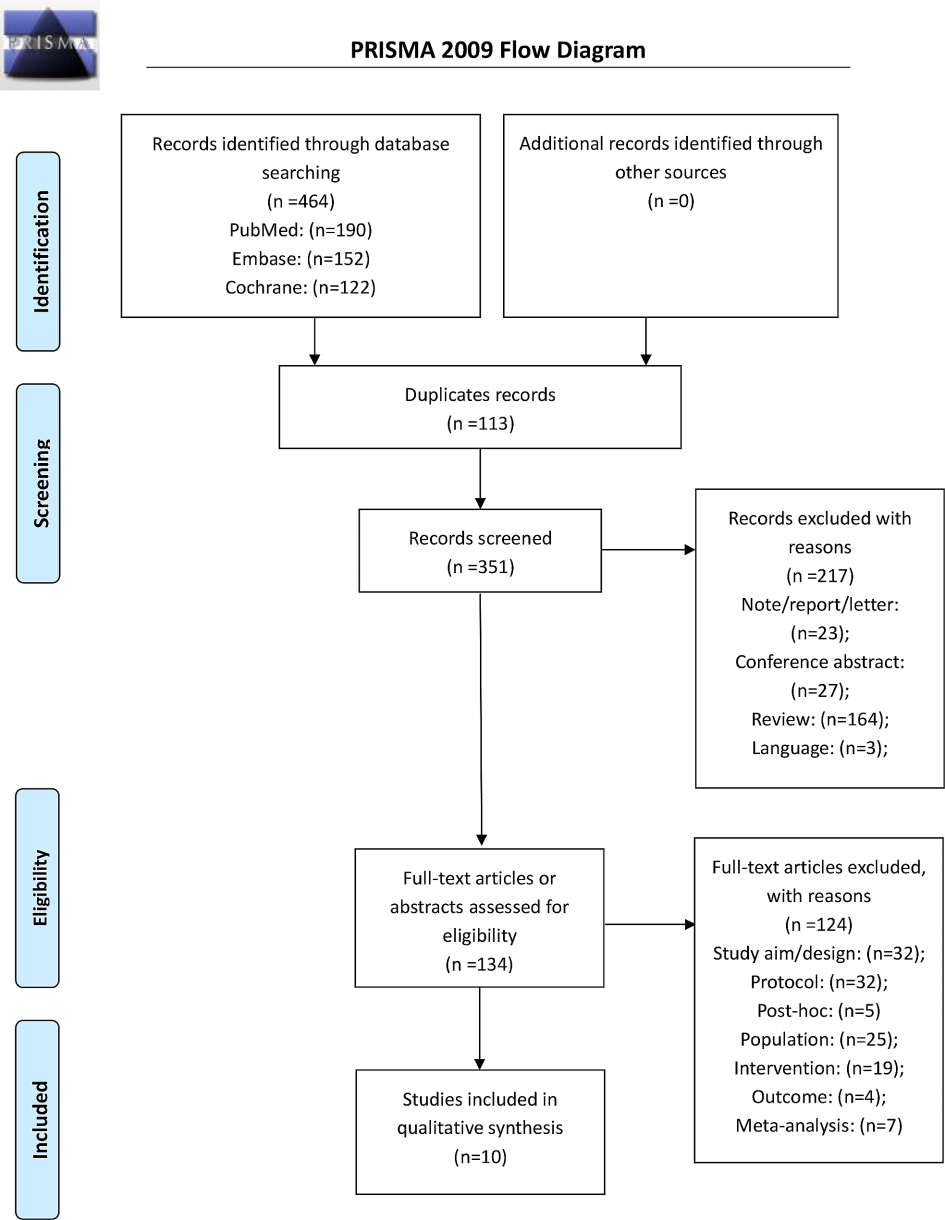
PRISMA 2009 Flow Diagram

The literature searched retrieved 464 records (Fig. 1). After removing the duplicates, 351 records were screened, and 217 were excluded. Among the exclude articles, 27 were conference abstract, 164 were reviews, 23 were note/report/letter, and three articles were not published in English. Among the remaining 134 full-text articles assessed for eligibility, 244 were excluded because of study aim (*n* = 32), population (*n* = 25), study type (*n* = 37), intervention (*n* = 19), outcome (*n* = 4), and meta-analysis (*n* = 7).

Therefore, 10 studies (11 datasets) were included in this meta-analysis (Table 1) [10, 15–18, 28–32]. Those 10 studies included 1187 children. Patient age varied from 5 to 17 years. The type of delivery of intervention included in-person access, web-based, and phone-based. CBT protocols vary among studies (Table 2), as well as the measured outcomes (Table 3).

**Table 1:** Literature search and study characteristic

Author, year	Country	Patients	Treatment		Type of Intervention	Duration	Outcome	Assessments	Sample size		Gender
			Intervention	Control					Intervention	Control	
Cunningham, 2022	USA	9–14 y	CBT + TAU	TAU	In-person + Web-based	6 sessions, 8 weeks	FDI, VAS, SCARED, CDI	T0: baseline T1: 8 weeks	40	39	32 m, 47 f
Duarte, 2006	Brazil	5–14 y	CBT + TAU	TAU	In-person	4 sessions, 4 months	VAS	After each session	15	17	10 m, 22 f
Grob, 2013	Germany	7–12 y	CBT	TAU	In-person	6 sessions, 6 weeks	KINDL-R, VAS, PedsQL	T0: baseline T1: after treatment T2: 3 months	15	14	4 m, 25 f
Levy, 2010	USA	7–17 y	CBT + TAU	Education + TAU	In-person	3 sessions, 3 weeks	FPSR, CSI, FDI, CDI, PRI	T0: baseline T1: after treatment T2: 3 months T3: 6 months	100	100	55 m, 145 f
Levy, 2017a	USA	7–12 y	CBT + TAU	Education + TAU	In-person	3 sessions, 3 weeks	API, FDI, PBCL, CSI, PedsQL, PRI	T0: baseline T1: 1 week after treatment T2: 3 months T3: 6 months	107	55	112 m, 204 f
Levy, 2017b	USA	7–12 y	CBT + TAU	Education + TAU	Phone	3 sessions, 3 weeks	API, FDI, PBCL, CSI, PedsQL, PRI	T0: baseline T1: 1 week after treatment T2: 3 months T3: 6 months	100	54	
Palermo, 2009	USA	11–17 y	CBT + TAU	TAU	Web-based	6 sessions, 6 weeks	CALI, NRS, RCADS	T0: baseline T1: 3-month	26	22	13 m, 35 f
Palermo, 2016	USA	11–17 y	CBT + TAU	Education + TAU	Web-based	8 sessions, 8 weeks	CALI, NRS, BAQP	T0: baseline T1: after treatment T2: 6-month	138	135	68 m, 205 f
Robins, 2005	USA	6–16 y	CBT + TAU	TAU	In-person	5 sessions, 10 months	API, CSI, FDI	T0: baseline T1: after treatment	40	29	30 m, 39 f
Van der veek, 2013	Netherlands	8–17 y	CBT	Intensive medical care	In-person	6 sessions, 6 weeks	API, CSI, FDI, RCADS, KIDSCREEN- 27, ADIS	T0: baseline T1: 2 weeks after treatment T2: 6 months	52	52	29 m, 75 f
Warner, 2011	USA	8–16 y	CBT	TAU	In-person	12 sessions, 10 weeks	Self-reported pain scale, CSI	T0: baseline T1: after treatment	20	17	14 m, 26 f

CBT: cognitive behavior treatment, TAU: treatment as usual, FDI: functional disability inventory, VAS: visual analog scale, SCARED: Screen for child anxiety related disorders, API: abdominal pain index, FPSR: face pain scale-revised, PedsQL: Pediatric quality of life inventory, PRI: Pain response inventory, CDI: Children’s depression inventory, CALI: child activity limitations interview, NRS: numerical rating scale, BAPQ: Bath Adolescent Pain Questionnaire, RCADS: Revised child anxiety and depression scale, ASWS: adolescent sleep wake scale, CSI: child somatization inventory, PBCI: Pain behavior check list, MASC: multidimensional anxiety scale for children

**Table 2:** Description of the cognitive and behavior intervention

Author	Description of the cognitive and behavior intervention
Cunningham, 2022	Teach evidence-based cognitive behavioral strategies to cope with pain and anxiety, given that anxiety commonly co-occurs in this population and is predictive of poor outcome.
Duarte, 2006	Modify inadequate responses of the child reacting to pain crises and the response of others, minimizing poorly adaptive and maximizing well-adapted behaviors toward pain; Physical exercise-walks, swimming, cycling, running around the block or the home, shadow boxing. Relaxation-breathing exercises and muscle relaxation with the objective of minimizing sympathetic nervous system activity during pain crises. Thought-stopping-with the objective of reducing anxiety. Distraction and attention-to-distract the patient when pain starts, redirecting their attention far from the pain and thus attenuating the neuronal impulses invoked by pain. Imagination-to encourage the child to think of pleasant or exciting situations when confronted with pain.
Grob, 2013	Imparting knowledge and teaching coping strategies, relaxation technique training, identification and change of negative pain-related thoughts and attention bias, techniques for increasing self-esteem.
Levy, 2010	Relaxation training, responses to illness and wellness behaviors, and cognitive restructuring to address and alter dysfunctional cognitions regarding symptoms and their implications for functioning.
Levy, 2017	Teaching parents to differentially attend to and reinforce wellness behaviors (those behaviors incompatible with illness and disability) while decreasing attention and reinforcement of illness behaviors related to abdominal pain; to use more adaptive cognitive coping strategies including reducing catastrophizing cognitions and threat appraisals regarding FAP; and to model healthy responses to somatic symptoms.
Morris, 2021	recognizing stress in their child, using operant strategies to change child behavior, modeling of adaptive coping behaviors, sleep hygiene, and parent-child communication.
Palermo, 2009	education about chronic pain, recognizing stress and negative emotions, deep breathing and relaxation, distraction, cognitive skills, sleep hygiene and lifestyle, staying active, and relapse prevention
Palermo, 2016	education about chronic pain, recognizing stress and negative emotions, deep breathing and relaxation, implementing coping skills at school, cognitive skills (e.g., reducing negative thoughts), sleep hygiene and lifestyle, staying active (e.g., activity pacing, pleasant activity scheduling), and relapse prevention.
Robins, 2005	Develop understanding of child’s pain; Increase repertoire of pain management techniques; Increase understanding of connection between stress and pain perception; Increase repertoire of pain management techniques; Encourage child to “take control” of abdominal pain; Increase child’s awareness of positive and negative self-talk and impact on pain; Increase “partnership” between child and parent in active management of pain; Reinforce gains and prepare for continued coping.
Van der veek, 2013	Relaxation training; Cognitive therapy; Behavior therapy directed at behavior child; Behavior therapy directed at behavior of parents.
Warner, 2011	Applying relaxation, cognitive restructuring and exposure exercises to target fears related to physical pain and anxiety-inducing situations.

**Table 3:** Assessment tools for the outcome measurements

	Measurement	Study
Pain related inventory		
FDI: functional disability inventory	Pain-related disability	Cunningham, 2022; Levy, 2010; Levy, 2017; Robins, 2005; Van der veek, 2013
VAS: visual analog scale	Pain intensity	Cunningham, 2022; Duarte, 2006; Grob, 2013
NRS: numerical rating scale	Pain intensity	Palermo, 2009; Palermo, 2016
API: abdominal pain index	Pain severity	Cunningham, 2022; Levy, 2017; Robins, 2005; Van der veek, 2013
Self-reported pain scale	Pain severity	Warner 2011
KINDL-R	Pain-related impairment	Grob, 2013
FPSR: face pain scale-revised	Pain intensity	Levy, 2010
PRI: Pain response inventory	Pain coping skill	Levy, 2010; Levy, 2017
CALI: Child Activity Limitations Interview	Activity limitations	Palermo, 2009; Palermo, 2016
Depression/anxiety		
CDI: Children’s depression inventory	Depression	Cunningham, 2022; Levy, 2010
SCARED: Screen for child anxiety related disorders	Anxiety	Cunningham, 2022
RCADS: Revised child anxiety and depression scale	Anxiety and depression	Palermo, 2009; Van der veek, 2013
BAPQ: Bath Adolescent Pain Questionnaire	Anxiety and depression	Palermo, 2016
Gastrointestinal symptoms		
CSI: child somatization inventory	Gastrointestinal symptom severity	Levy, 2010; Levy, 2017; Robins, 2005; Van der veek, 2013; Warner 2011
Quality of life		
PedsQL: Pediatric quality of life inventory,	Quality of life	Grob, 2013; Levy, 2017
KIDSCREEN-27	Quality of life	Van der veek, 2013

### Quality of all included studies

Among the 10 randomized controlled trials, the overall risk of bias was low in 8 studies [10, 16, 17, 28–30]. Some concerns were raised in the assessment for bias arising from the randomization process in one study [15], and bias due to deviations from intended interventions in three studies [15, 17, 31]. And one study might be biased due to missing outcome data [31]. All 10 studies were graded as low risk of bias regarding the terms of selection of the reported result and the measurement of outcomes.

### Effect of CBT on FDI

**Fig. 2:**
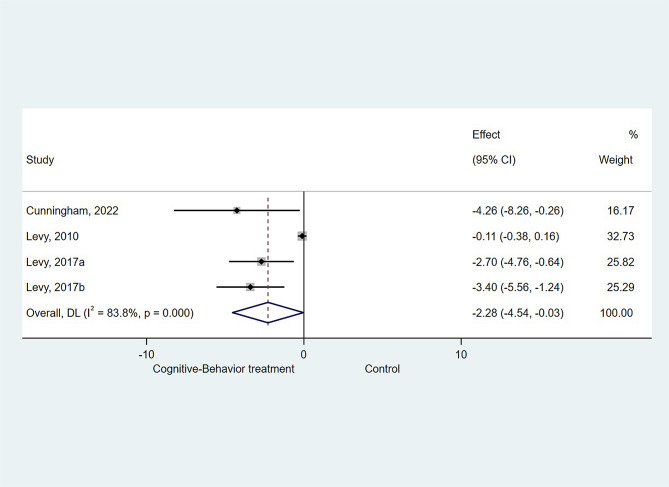
Forest plot of functional disability inventory: Cognitive-Behavior treatment vs. Control

Three studies (4 datasets) could [10, 28, 29] be included for the meta-analysis of CBT on FDI. The results showed that CBT resulted in better improvement in FDI compared with the control groups (SMD=-2.282, 95%CI: -4.537 to -0.027, *P* = 0.047; I^2^ = 83.8%, P_heterogeneity_<0.001, Fig. 2).

### Effect of CBT on Pain intensity

**Fig. 3:**
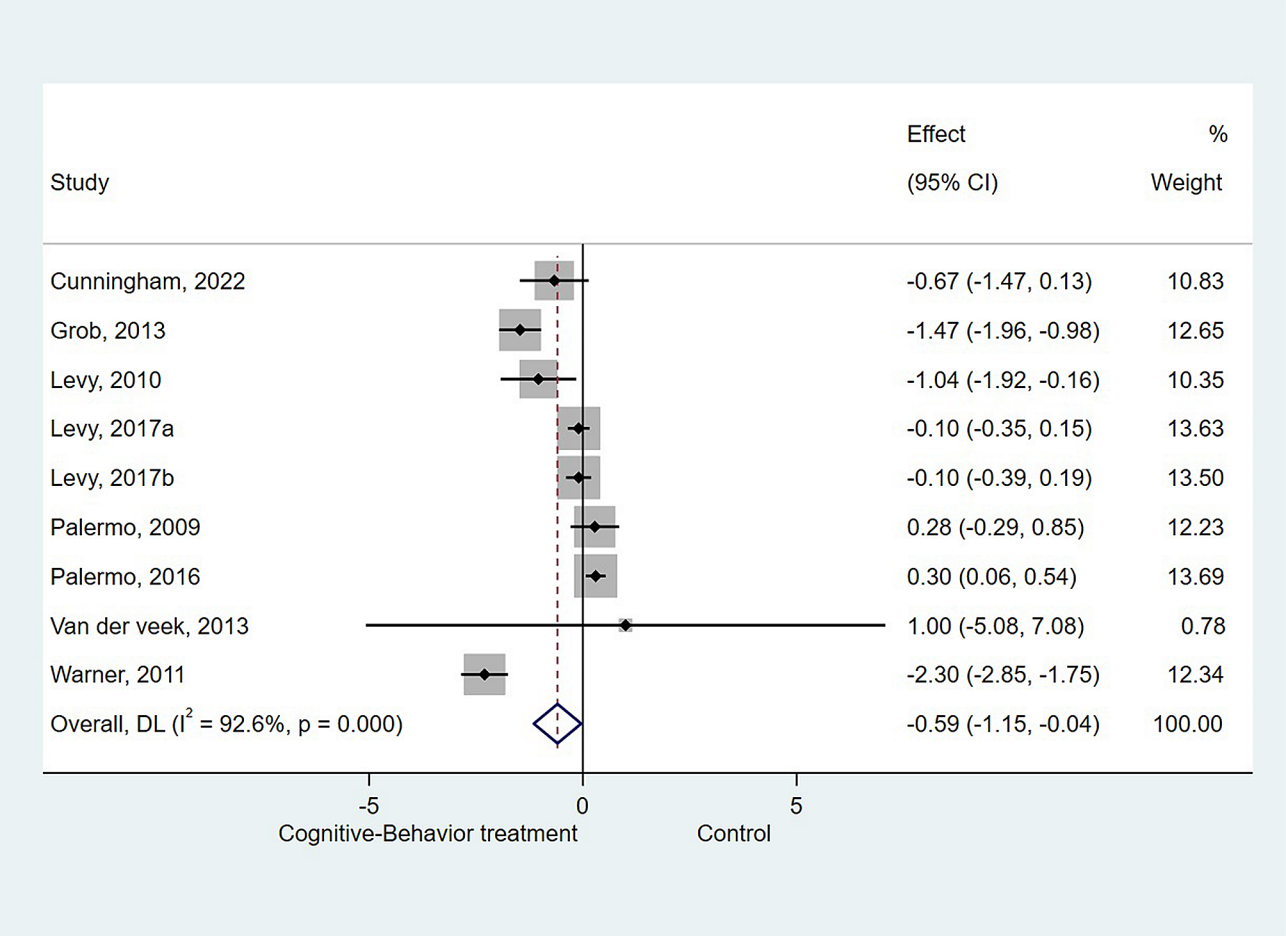
Forest plot of pain intensity: Cognitive-Behavior treatment vs. Control

Eight studies (nine datasets) [10, 16–18, 28–30, 32] were included for the meta-analysis of CBT on pain intensity. The results showed that CBT resulted in a significant effect on ameliorating pain intensity compared with the control groups (SMD=-0.594, 95%CI: -1.147 to -0.040, *P* = 0.036; I^2^ = 92.6%, P_heterogeneity_<0.001, Fig. 3).

### Effect of CBT on children’s depression and anxiety

**Fig. 4:**
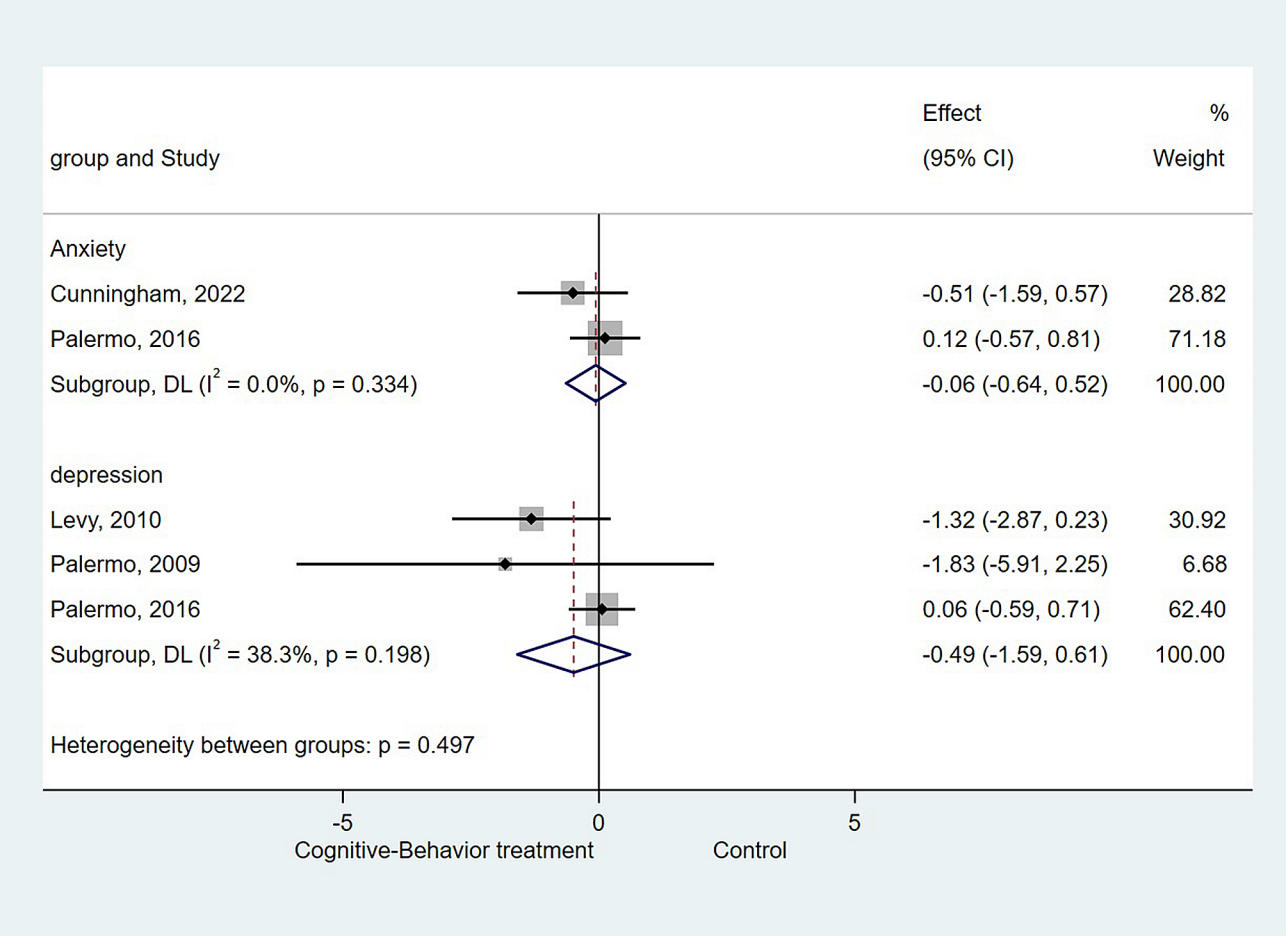
Forest plot of depression and anxiety: Cognitive-Behavior treatment vs. Control

Three studies [10, 16, 30] examining the effect of CBT applied on the severity of children’s depression showed no significant difference compared to usual treatment (SMD=-0.493, 95%CI: -1.594 to 0.608, *P* = 0.380; I^2^ = 38.3%, P_heterogeneity_=0.198). And two studies [28, 30] examining the effect of CBT applied on the severity of children’s anxiety also showed no significant difference compared to usual treatmen (SMD=-0.062, 95%CI: -0.640 to 0.517, *P* = 0.835; I^2^ = 0%, P_heterogeneity_=0.334, Fig. 4).

### Effect of CBT on gastrointestinal symptoms

**Fig. 5:**
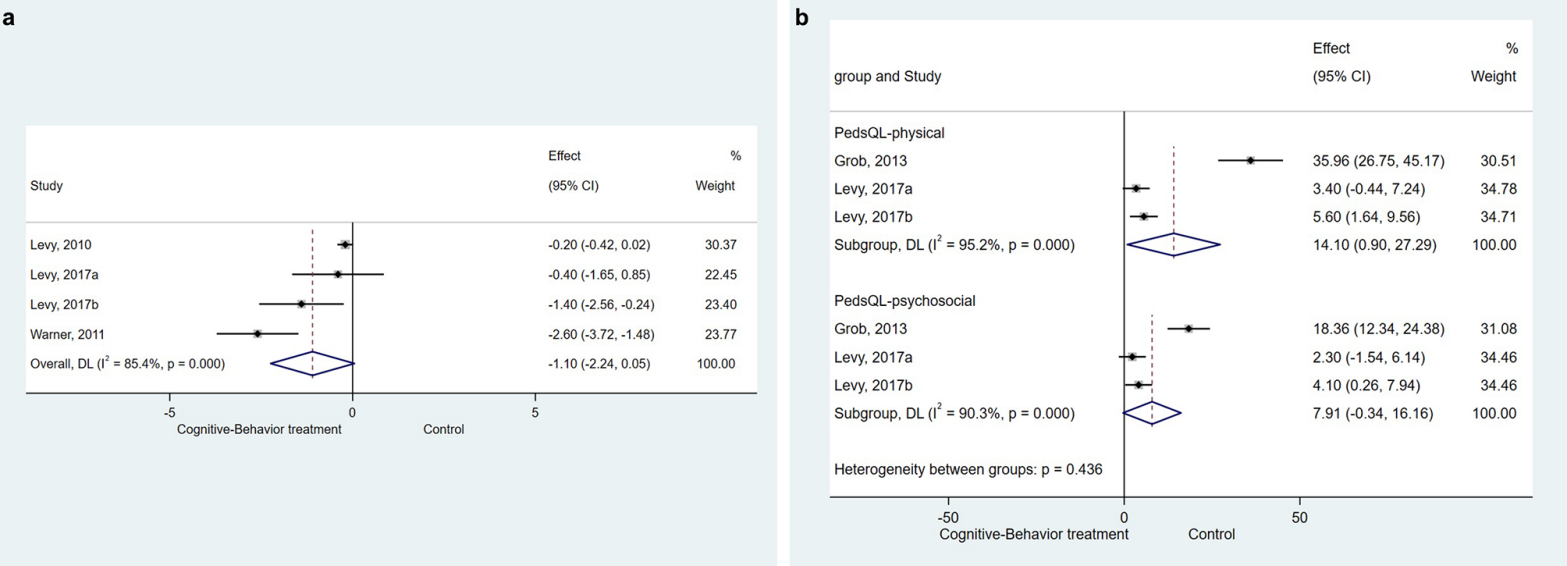
(**a**) Forest plot of gastrointestinal symptoms: Cognitive-Behavior treatment vs. Control. (**b**) Forest plot of Quality of life: Cognitive-Behavior treatment vs. Control

Three studies (four datasets) [10, 29, 32] were included for the meta-analysis of CBT on gastrointestinal symptoms. The results showed that CBT resulted in a comparable effect compared with the control groups (SMD=-1.096 95%CI: -2.243 to 0.050, *P* = 0.061; I^2^ = 85.4%, P_heterogeneity_<0.001, Fig. 5a).

### Effect of CBT on quality of life

Two studies (three datasets) [17, 29] reported the post-treatment differences between CBT and control in the quality of life regarding the physical and psychological terms. The results showed a significant differences in child physical quality of life from the comparation of CBT and control (SMD = 14.097, 95%CI: 0.901 to 27.292, *P* = 0.036; I^2^ = 95.2%, P_heterogeneity_<0.001), while no significant difference was found in child psychological quality of life between children in CBT group and control group (SMD = 7.912, 95%CI: -0.338 to 16.163, *P* = 0.060; I^2^ = 90.3%, P_heterogeneity_<0.001, Fig. 5b).

### Sensitivity analysis

The results of sensitivity analysis conducted by omitting one study at a time were similar in the combined results of FAP, pain intensity, and gastrointestinal symptoms, without great fluctuation, suggesting that the pooled SWDs were relatively stable (Additional file 2–4).

## Discussion

This study was designed to systematically review the literature for RCTs that investigated the efficacy of CBT in children with FAP. We used data from 10 randomized controlled trials including 1187 children aged 5–17 with FAP. We observed differences in post-treatment FAP and pain intensity for children receiving CBT compared with children receiving treatment as usual. CBT may be considered as validated non-pharmacological treatment for chronic functional abdominal pain in pediatric patients.

A previous meta-analysis investigating the effectiveness of CBT in the treatment of chronic pain in children also showed similar results [33]. Despite only 5 RCTs were included in this previous meta-analysis, the authors concluded that CBT may be effective in reducing child reported pain symptomology. Compared to our meta-analysis, aside from the extreme distinction regarding the number of RCTs and patients involved, one major difference is that they failed to synthesis the quantitative results from the included studies due to the lack of data from posted results. Luckily in our analysis, as more researchers investigating the effect of CBT among children with FAP, we were able to collect more data into our analysis. Moreover, as emerging evidence support the positive efficacy of CBT in pain control [12], we sought to assess multiple, relevant outcome domains that are related to the CBT including physical functioning (activity limitations), emotional functioning (anxiety or depressive symptoms), and the quality of life.

The presence of anxiety in conjunction with FAP is quite common [34–37]. Evidence has suggested that anxiety is associated with increased pain and disability [38]. When investigating severity of depression and anxiety of patients, we observed that the CBT seemed to be comparable in reducing the symptoms of depression and anxiety symptoms compared to the usual treatment, and the results from individual studies were extensively consistent. One possible theory for this result is that CBT for pain management does not always directly target anxiety. Thus, the CBT intervention, which targets pain and anxiety when appropriate, has the potential to substantially improve patient outcomes. Based on the study findings, it may be feasible for patients with FAP to receive direct relaxation training, coping skills training, and psychoeducation for pain management as part of standard medical treatment. In terms of the quality of life, though only two studies were included for this analysis, CBT seems to have better effect in improving children’s physical and psychological quality of life compared to usual treatment. Similar to our results, previous research suggested a causal link between pain and quality of life and encouraged intervention targeting the children’s cognitive coping strategies, problem solving, and positive self-statements as they found that pain can be mediated by psychosocial behavior [39].

FAP in children has been defined variably across different diagnostic criteria, reflecting an evolving understanding of this condition. The Apley’s criteria [40], one of the earliest, conceptualized FAP as recurrent abdominal pain for at least three months, without physical cause, in children aged 4–16 years. Subsequently, the Rome criteria provided more structured definitions. Rome II criteria (1999) emphasized symptom duration and frequency, while Rome III (2006) and Rome IV (2016) offered more detailed classifications, including subcategories like Functional Abdominal Pain Syndrome and Irritable Bowel Syndrome [41, 42]. This nuanced categorization underscores the importance of recognizing alarm signs indicative of organic abnormalities and acknowledges the complex interplay of psychosocial, genetic, and environmental factors through the gut-brain axis in FAP. It is crucial to acknowledge the broad span of publication years of the included studies, which has led to the incorporation of data on FAP in children diagnosed under varying criteria. This temporal diversity encompasses significant shifts in diagnostic approaches, from the earlier Apley’s criteria to the more recent and detailed Rome II, III, and IV criteria. Such evolution in diagnostic standards reflects a deepening understanding of FAP, but also introduces heterogeneity in our analysis. This variance in diagnostic criteria over time could potentially impact the comparability of study outcomes and interpretations of the efficacy of CBT for FAP. For instance, studies based on earlier criteria might have included children with a wider range of symptoms, possibly diluting the perceived effectiveness of CBT in more narrowly defined FAP subtypes as per later criteria. Additionally, the evolving understanding of the role of psychological factors in FAP, such as anxiety and depression, and their impact on treatment outcomes, further complicates direct comparisons across studies [19]. Furthermore, with the broadening understanding of FAP, especially regarding the gut-brain axis and psychosocial contributors, different treatment strategies combined with CBT were comprehensively evolved to target not just the physical symptoms but also the psychological aspects, which might introduce bias for evaluating the efficacy of CBT in our analysis. Therefore, while our meta-analysis provides valuable insights into the effectiveness of CBT for FAP in children, these findings should be interpreted considering the heterogeneity of the diagnostic criteria and detailed treatment strategies used across the included studies. This diversity underscores the need for cautious interpretation and suggests a potential avenue for future research to explore the efficacy of CBT within the context of more uniformly defined FAP subtypes.

The conclusions of the present meta-analysis must be considered along with its limitations. The outcome measurement scales and the protocol of the cognitive-behavior treatment varies among the RCTs. This bias was attenuated with the use of SMD and random-effects modeling, but potential bias may still influence the results. Second, different interventions (with or without usual care) were combined together for analysis, which might introduce bias or dilute the observations. Third, the patients in the different RCTs were treated with different number of sessions and time log, and the effects of CBT were assessed at different times following treatment. Nevertheless, the sensitivity analysis showed a robust effect of CBT in children with FAP. Fourth, some studies did not report or not appropriately report the mean differences and standard deviation from baseline to follow-up visits, therefor, in order to analyze the results, we had to exclude those studies from the quantitative analyses. Despite we tried our best to estimate some of the parameters based on what was given in the original articles, which was per suggested according to the Cochrane Handbook, but this could still introduce bias. Finally, we failed to conducted subgroup analyses to compare the difference between in-person training with web-based training, which in our hypothesize might introduce potentially distinctive differences in terms of the outcomes, as we hypothesize that the web-based training overlooked the interactivity, personalization, and communication possible with the patients, and instead relied heavily on web-based or telephone multimedia-formatted interaction with a therapist, thus limiting the potential dissemination of the intervention. Researchers are encouraged to design future trials that applying different type of CBT and to incorporate standard outcome measures. Additional research remains to be completed for CBT in children with FAP. Furthermore, it is possible that the degree of caregiver involvement required may vary based on the severity of child/caregiver symptoms [43, 44]. Accordingly, additional work is necessary to investigate whether caregiver involvement can be effective for the reductions of pain in children.

## Conclusions

This meta-analysis showed a significant overall improvement in ameliorating FAP, pain intensity, and physical QoL for children with FAP after pooling the results from 10 recent RCTs that examined CBT intervention. Despite no significant differences were observed in the assessments of depression or anxiety, and gastrointestinal symptoms between CBT and the usual treatment, CBT in the setting of FAP demonstrates promising developments and highlights the need for future research.

### Electronic supplementary material

Below is the link to the electronic supplementary material.

Supplementary Material 1

Supplementary Material 2

Supplementary Material 3

Supplementary Material 4

## Data Availability

All data generated or analysed during this study are included in this published article and its supplementary information files.

## Abbreviations

API: Abdominal Pain Index
BAPQ: Bath Adolescent Pain Questionnaire
CBT: Cognitive-Behavior Therapy
CDI: The Children’s Depression Inventory
CIs: Confidence intervals
CSI: The Children Somatization Inventory
FAP: Functional abdominal pain
FDI: Functional disability inventory
FPSR: Faces Pain Scale-Revised
NRS: Numerical Rating Scale
PedsQL: Pediatric Quality of Life Inventory
RCADS: Revised Child Anxiety and Depression Scale
SMD: Standardized mean difference
VAS: Visual Analog Scale

## References

[1] Rasquin A, Di Lorenzo C, Forbes D, Guiraldes E, Hyams JS, Staiano A (2006). Childhood functional gastrointestinal disorders: child/adolescent. Gastroenterology.

[2] Korterink JJ, Diederen K, Benninga MA, Tabbers MM (2015). Epidemiology of pediatric functional abdominal pain disorders: a meta-analysis. PLoS ONE.

[3] Drossman DA (2016). Functional gastrointestinal disorders: history, pathophysiology, clinical features and Rome IV. Gastroenterology.

[4] Koppen IJ, Nurko S, Saps M, Di Lorenzo C, Benninga MA (2017). The pediatric Rome IV criteria: what’s new?. Expert Rev Gastroenterol Hepatol.

[5] Shelby GD, Shirkey KC, Sherman AL, Beck JE, Haman K, Shears AR (2013). Functional abdominal pain in childhood and long-term vulnerability to anxiety disorders. Pediatrics.

[6] Gieteling MJ, Bierma-Zeinstra SM, Passchier J, Berger MY (2008). Prognosis of chronic or recurrent abdominal pain in children. J Pediatr Gastroenterol Nutr.

[7] van Tilburg MA, Felix CT (2013). Diet and functional abdominal pain in children and adolescents. J Pediatr Gastroenterol Nutr.

[8] van Tilburg MAL, Carter CA (2018). Integration of Biomedical and Psychosocial treatments in Pediatrics Functional Gastrointestinal disorders. Gastroenterol Clin North Am.

[9] Abbott RA, Martin AE, Newlove-Delgado TV, Bethel A, Thompson-Coon J, Whear R (2017). Psychosocial interventions for recurrent abdominal pain in childhood. Cochrane Database Syst Rev.

[10] Levy RL, Langer SL, Walker LS, Romano JM, Christie DL, Youssef N (2010). Cognitive-behavioral therapy for children with functional abdominal pain and their parents decreases pain and other symptoms. Am J Gastroenterol.

[11] Bonnert M, Olén O, Lalouni M, Benninga MA, Bottai M, Engelbrektsson J (2017). Internet-delivered cognitive behavior therapy for adolescents with irritable bowel syndrome: a Randomized Controlled Trial. Am J Gastroenterol.

[12] Lalouni M, Ljótsson B, Bonnert M, Ssegonja R, Benninga M, Bjureberg J (2019). Clinical and cost effectiveness of online cognitive behavioral therapy in Children with Functional Abdominal Pain disorders. Clin Gastroenterol Hepatol.

[13] Eccleston C, Palermo T, Williams Lewandowski A, Morley S (2014). Psychological therapies for the management of chronic and recurrent pain in children and adolescents (review). Cochrane Collab.

[14] Carter BD, Threlkeld BM (2012). Psychosocial perspectives in the treatment of pediatric chronic pain. Pediatr Rheumatol Online J.

[15] Duarte MA, Penna FJ, Andrade EM, Cancela CS, Neto JC, Barbosa TF (2006). Treatment of nonorganic recurrent abdominal pain: cognitive-behavioral family intervention. J Pediatr Gastroenterol Nutr.

[16] Palermo TM, Wilson AC, Peters M, Lewandowski A, Somhegyi H (2009). Randomized controlled trial of an internet-delivered family cognitive-behavioral therapy intervention for children and adolescents with chronic pain. Pain.

[17] Groß M, Warschburger P (2013). Evaluation of a cognitive-behavioral pain management program for children with chronic abdominal pain: a randomized controlled study. Int J Behav Med.

[18] van der Veek SM, Derkx BH, Benninga MA, Boer F, de Haan E (2013). Cognitive behavior therapy for pediatric functional abdominal pain: a randomized controlled trial. Pediatrics.

[19] Saini S, Narang M, Srivastava S, Shah D (2021). Behavioral intervention in children with Functional Abdominal Pain disorders: a promising option. Turk J Gastroenterol.

[20] Higgins JP, Thomas J, Chandler J, Cumpston M, Li T, Page MJ, et al. Cochrane handbook for systematic reviews of interventions. John Wiley & Sons; 2019. 10.1002/14651858.ED000142 PMC10284251 31643080

[21] Selçuk AA (2019). A guide for systematic reviews: PRISMA. Turk Arch Otorhinolaryngol.

[22] Aslam S, Emmanuel P (2010). Formulating a researchable question: a critical step for facilitating good clinical research. Indian J Sex Transm Dis AIDS.

[23] Walker LS, Greene JW (1991). The functional disability inventory: measuring a neglected dimension of child health status. J Pediatr Psychol.

[24] Kashikar-Zuck S, Flowers SR, Claar RL, Guite JW, Logan DE, Lynch-Jordan AM (2011). Clinical utility and validity of the functional disability inventory among a multicenter sample of youth with chronic pain. Pain.

[25] Sterne JAC, Savović J, Page MJ, Elbers RG, Blencowe NS, Boutron I (2019). RoB 2: a revised tool for assessing risk of bias in randomised trials. BMJ.

[26] Hozo SP, Djulbegovic B, Hozo I (2005). Estimating the mean and variance from the median, range, and the size of a sample. BMC Med Res Methodol.

[27] Pustejovsky JE, Rodgers MA (2019). Testing for funnel plot asymmetry of standardized mean differences. Res Synth Methods.

[28] Cunningham NR, Kalomiris A, Peugh J, Farrell M, Pentiuk S, Mallon D (2021). Cognitive behavior therapy tailored to anxiety symptoms improves Pediatric Functional Abdominal Pain outcomes: a Randomized Clinical Trial. J Pediatr.

[29] Levy RL, Langer SL, van Tilburg MAL, Romano JM, Murphy TB, Walker LS (2017). Brief telephone-delivered cognitive behavioral therapy targeted to parents of children with functional abdominal pain: a randomized controlled trial. Pain.

[30] Palermo TM, Law EF, Fales J, Bromberg MH, Jessen-Fiddick T, Tai G (2016). Internet-delivered cognitive-behavioral treatment for adolescents with chronic pain and their parents: a randomized controlled multicenter trial. Pain.

[31] Robins PM, Smith SM, Glutting JJ, Bishop CT (2005). A randomized controlled trial of a cognitive-behavioral family intervention for pediatric recurrent abdominal pain. J Pediatr Psychol.

[32] Warner CM, Colognori D, Kim RE, Reigada LC, Klein RG, Browner-Elhanan KJ (2011). Cognitive-behavioral treatment of persistent functional somatic complaints and pediatric anxiety: an initial controlled trial. Depress Anxiety.

[33] Lonergan A (2016). The effectiveness of cognitive behavioural therapy for pain in childhood and adolescence: a meta-analytic review. Ir J Psychol Med.

[34] Cunningham NR, Cohen MB, Farrell MK, Mezoff AG, Lynch-Jordan A, Kashikar-Zuck S (2015). Concordant parent-child reports of anxiety predict impairment in youth with functional abdominal pain. J Pediatr Gastroenterol Nutr.

[35] Cunningham NR, Moorman E, Brown CM, Mallon D, Chundi PK, Mara CA (2018). Integrating psychological screening into Medical Care for Youth with Abdominal Pain. Pediatrics.

[36] Dufton LM, Dunn MJ, Compas BE (2009). Anxiety and somatic complaints in children with recurrent abdominal pain and anxiety disorders. J Pediatr Psychol.

[37] Lipsitz JD, Masia C, Apfel H, Marans Z, Gur M, Dent H (2005). Noncardiac chest pain and psychopathology in children and adolescents. J Psychosom Res.

[38] Wendland M, Jackson Y, Stokes LD (2010). Functional disability in paediatric patients with recurrent abdominal pain. Child Care Health Dev.

[39] Warschburger P, Hänig J, Friedt M, Posovszky C, Schier M, Calvano C (2014). Health-related quality of life in children with abdominal pain due to functional or organic gastrointestinal disorders. J Pediatr Psychol.

[40] Apley J, Naish N (1958). Recurrent abdominal pains: a field survey of 1,000 school children. Arch Dis Child.

[41] Spee LA, Lisman-Van Leeuwen Y, Benninga MA, Bierma-Zeinstra SM, Berger MY (2013). Prevalence, characteristics, and management of childhood functional abdominal pain in general practice. Scand J Prim Health Care.

[42] Demirören K, Güney B, Bostancı M, Ekici D (2022). A comparison between Rome III and Rome IV Criteria in Children with Chronic Abdominal Pain: a prospective Observational Cohort Study. Turk J Gastroenterol.

[43] Langer SL, Romano JM, Levy RL, Walker LS, Whitehead WE (2009). Catastrophizing and parental response to child Symptom complaints. Child Health Care.

[44] Langer SL, Walker LS, Romano JM, Whitehead WE, Feld L, Levy RL (2007). Predictors of maternal responses to child abdominal pain. Children’s Healthc.

