# Supplementary material for: Effect of cognitive-behavior therapy for children with functional abdominal pain: a meta-analysis
Source: BMC Gastroenterol. 2024 Feb 3;24:62. doi: 10.1186/s12876-024-03120-2 (PMC10838415; doi:10.1186/s12876-024-03120-2)
Supplement: Supplementary file 1 — Supplementary Material 1 [file 12876_2024_3120_MOESM1_ESM.docx]

Additional file 1. Cochrane risk of bias assessment tool 2 (ROB-2) for randomized controlled trial

| Study | 1 | 2 | 3 | 4 | 5 | 6 |
| --- | --- | --- | --- | --- | --- | --- |
| Cunningham, 2022 | Low | Low | Low | Low | Low | Low |
| Duarte, 2006 | Some concerns | Some concerns | Low | Low | Low | Some concerns |
| Grob, 2013 | Low | Some concerns | Low | Low | Low | Low |
| Levy, 2010 | Low | Low | Low | Low | Low | Low |
| Levy, 2017 | Low | Low | Low | Low | Low | Low |
| Palermo, 2009 | Low | Low | Low | Low | Low | Low |
| Palermo, 2016 | Low | Low | Low | Low | Low | Low |
| Robins, 2005 | Low | Some concerns | Some concerns | Low | Low | Some concerns |
| Van der veek, 2013 | Low | Low | Low | Low | Low | Low |
| Warner, 2011 | Low | Low | Low | Low | Low | Low |

1.Bias arising from the randomisation process; 2. Bias due to deviations from intended interventions; 3. Bias due to missing outcome data; 4.Bias in measurement of the outcome; 5. Bias in selection of the reported result; 6. Overall bias.
