# Supplementary figures and images for: Effect of cognitive-behavior therapy for children with functional abdominal pain: a meta-analysis
Source: BMC Gastroenterol. 2024 Feb 3;24:62. doi: 10.1186/s12876-024-03120-2 (PMC10838415; doi:10.1186/s12876-024-03120-2)

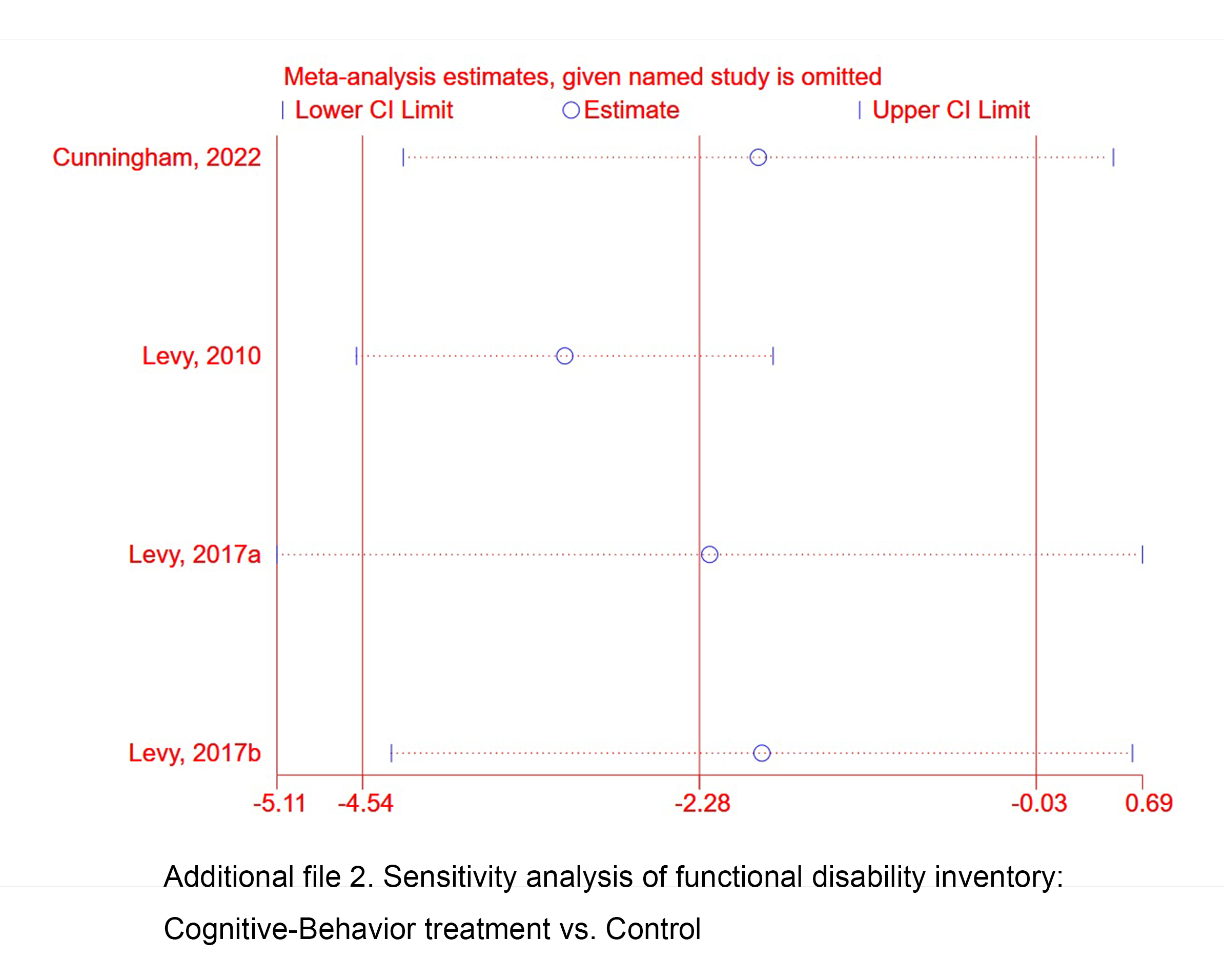

Supplement: Supplementary file 2 — Supplementary Material 2 [file 12876_2024_3120_MOESM2_ESM.png]

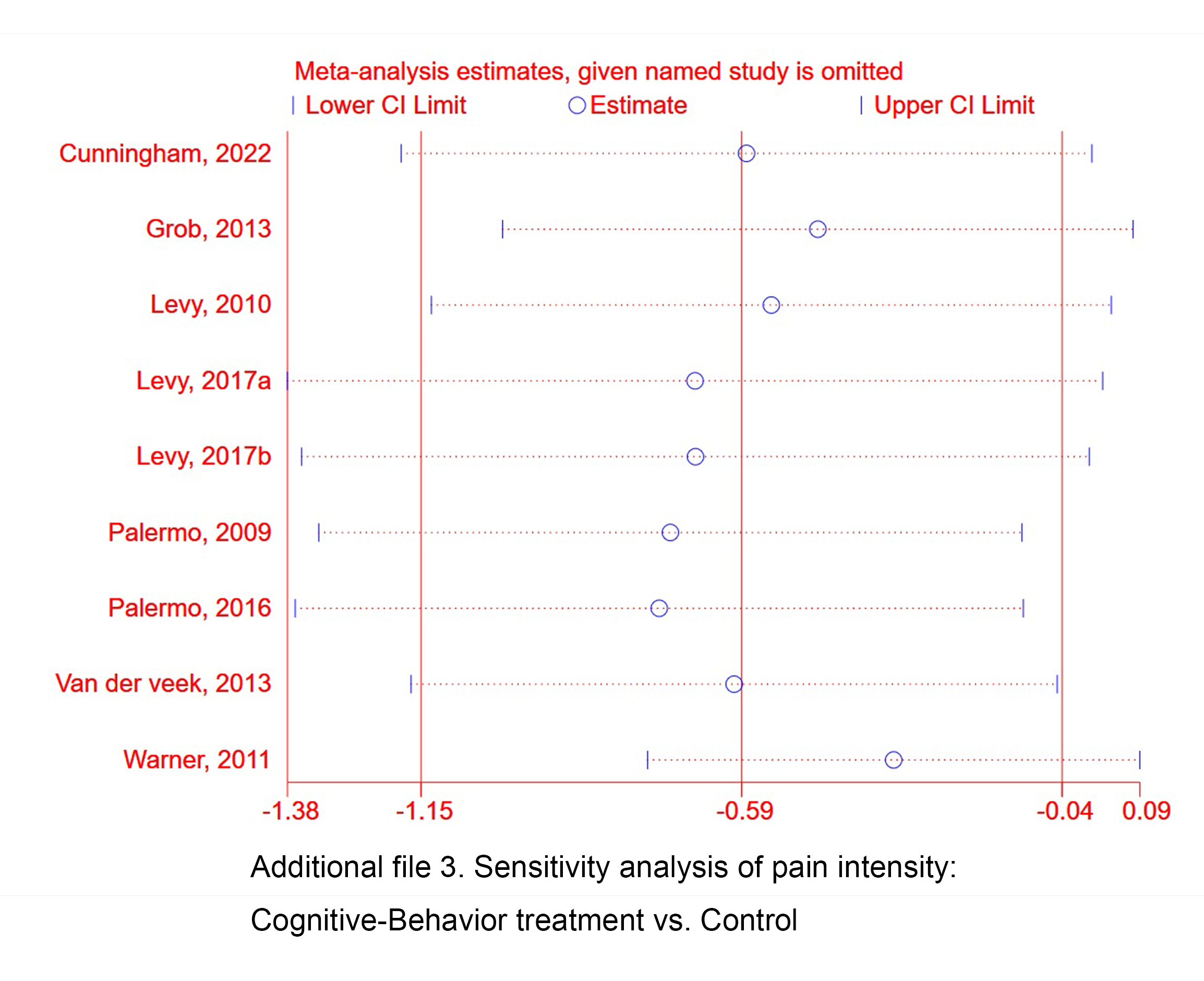

Supplement: Supplementary file 3 — Supplementary Material 3 [file 12876_2024_3120_MOESM3_ESM.png]

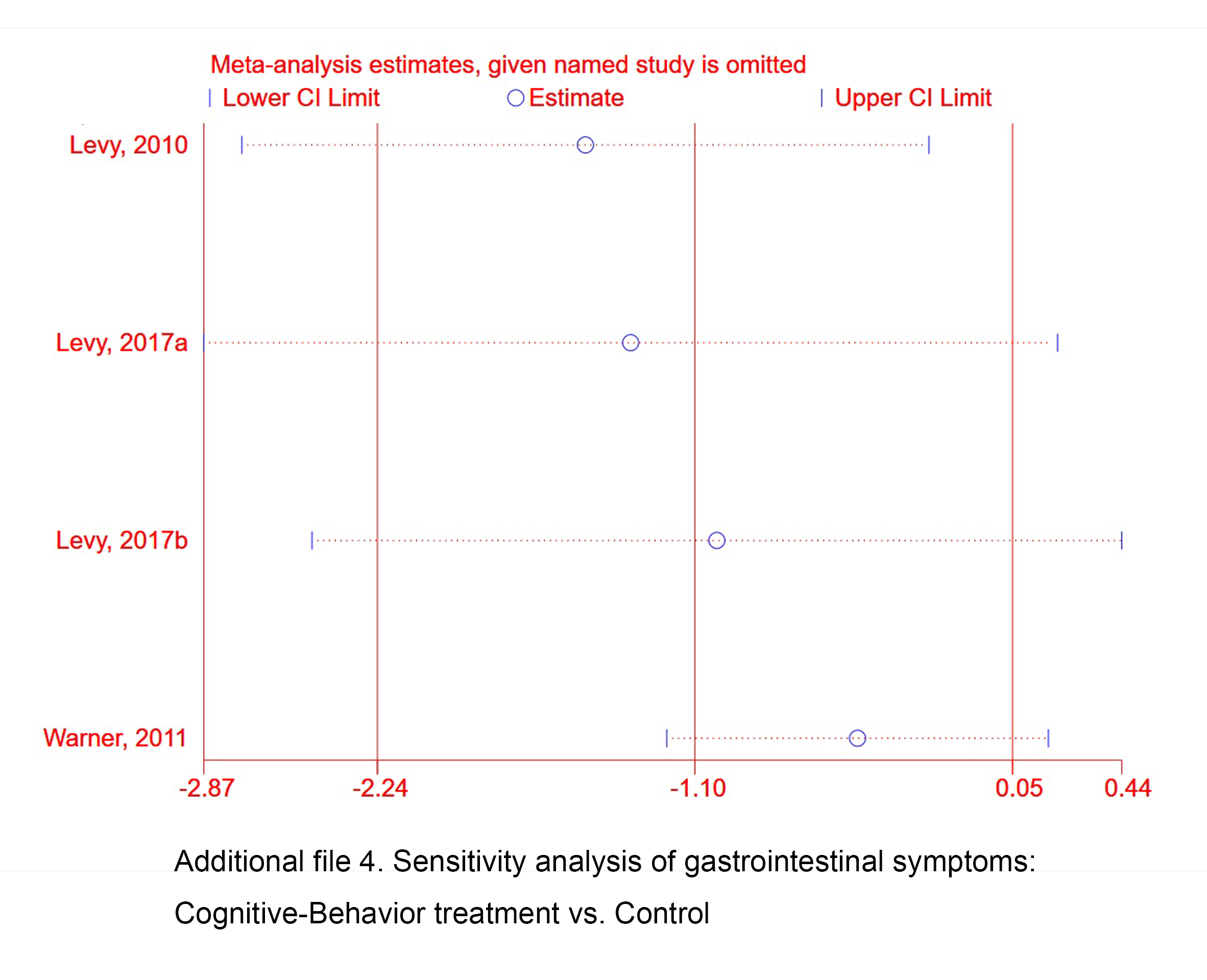

Supplement: Supplementary file 4 — Supplementary Material 4 [file 12876_2024_3120_MOESM4_ESM.png]
